# Wildlife uses and hunting patterns in rural communities of the Yucatan Peninsula, Mexico

**DOI:** 10.1186/1746-4269-8-38

**Published:** 2012-10-02

**Authors:** Dídac Santos-Fita, Eduardo J Naranjo, José Luis Rangel-Salazar

**Affiliations:** 1 Departamento de Ecología y Sistemática Terrestres, El Colegio de la Frontera Sur, Ap. 63, San Cristóbal de Las Casas, Chiapas 29290, México

**Keywords:** Maya, Mexico, Subsistence hunting, Wildlife, Yucatan

## Abstract

**Background:**

Subsistence hunting is a traditional practice providing food and many other goods for households in the Yucatan Peninsula, southeast Mexico. Economic, demographic, and cultural change in this region drive wildlife habitat loss and local extinctions. Improving our understanding about current practices of wildlife use may support better management strategies for conserving game species and their habitat. We aimed to evaluate if wildlife use remained relevant for the subsistence of rural residents of the Yucatan Peninsula, as well as if local hunting practices were related to environmental, geographical, and cultural factors.

**Methods:**

Fieldwork was done between March 2010 and March 2011. Information was obtained through conversations, interviews, and participant observation. Record forms allowed recording animals hunted, biomass extracted, distance intervals to hunting sites, habitat types and seasonality of wildlife harvests. Data were analyzed using one-way Analysis of Variance, and Generalized Linear Models.

**Results:**

Forty-six terrestrial vertebrate species were used for obtaining food, medicine, tools, adornments, pets, ritual objects, and for sale and mitigating damage. We recorded 968 animals taken in 664 successful hunting events. The Great Curassow, Ocellated Turkey, paca, white-tailed deer, and collared peccary were the top harvested species, providing 80.7% of biomass (10,190 kg). The numbers of animals hunted and biomass extracted declined as hunting distances increased from villages. Average per capita consumption was 4.65 ± 2.7 kg/person/year. Hunting frequencies were similar in forested and agricultural areas.

**Discussion:**

Wildlife use, hunting patterns, and technologies observed in our study sites were similar to those recorded in previous studies for rural Mayan and mestizo communities in the Yucatan Peninsula and other Neotropical sites. The most heavily hunted species were those providing more products and by-products for residents. Large birds such as the Great Curassow and the Ocellated Turkey were extremely important for local hunters, representing around 40% of total prey taken.

**Final considerations:**

Our results suggest that hunting is frequent in our study areas. Low human densities allow low hunting pressure on most game species and favor conservation of the tropical forest. We suggest that co-management may help regulating hunting, prioritizing cultural practices of sustainable use and conservation for benefiting local users and animal populations.

## Background

Wild animals have constituted a very significant element in human evolutionary history and culture around the world. Wildlife is a constantly subject of human use and management practices because of its multiple values, which in turn depend on each social group and specific historical and geographical context [1]. In rural tropical areas, a large proportion of human residents continue using a variety of wildlife species as sources of protein, fat, medicinal substances, clothes, tools, adornments, ritual objects, and income, among other purposes [2-6]. Most wildlife resources are obtained through hunting, considered a *subsistence* activity when its primary purpose is to satisfy the hunter’s and his family’s basic needs [7-9], and occasionally the whole community basic needs as well [10]. The main motivation for *commercial* hunters is to exchange their prey for money. In contrast, subsistence hunters usually go hunting for food, although the sale of surplus meat within their communities may occur [9].

Subsistence hunting frequently implies lower risks for wildlife populations than commercial hunting [11]. However, previous studies in areas suggest that subsistence practices increase pressure on hunted species, generally large and medium-sized vertebrates [12-17]. Among the wild terrestrial vertebrates providing food and other products to rural hunters in the Neotropics are dozens of mammals (ungulates, primates, armadillos, and large rodents), over 30 birds (mainly tinamous and cracids), and some reptiles (basically fresh-water turtles, iguanas and crocodiles) [9,18]. Overhunting on these species may induce severe decreases in their population sizes potentially leading to their local extinction, especially if they face habitat loss, degradation and fragmentation [12]. Indeed, overhunting, habitat loss and subsequent isolation may cause synergistic effects on tropical wildlife populations, driving them into the extinction vortex [19-21].

Recent studies have documented uses of over 60 species of wild mammals, birds and reptiles by indigenous and mestizo inhabitants in southeast Mexico [22]. Some of those studies have also described hunting patterns, trends and preferences, analyzing a wide array of involved ecological, social, and economic factors (Escamilla et al. [23] in Campeche; Jorgenson [7,8], Ramírez-Barajas and Naranjo [24] in Quintana Roo; Montiel et al. [10], Mandujano and Rico-Gray [25], Delfín and Chablé [26] in Yucatan; March [27], Naranjo [28] in Chiapas; Del Campo [29] in Oaxaca; and Reyes [30] in Tabasco, among others). Some studies on peninsular and Lacandon Maya groups have registered the beliefs and other cultural and religious elements related to hunting [31-33]. Other studies have focused on the relationship between hunting and agriculture (*garden-hunting* model) in Maya rural settlements of the Yucatan Peninsula [7,34]. Finally, a few more investigations in southeast Mexico have focused on assessing the impact of hunting on prey populations in tropical forests by: a) comparing harvest rates and abundance of prey populations in areas with different hunting pressure [35,36]; and b) evaluating hunting sustainability through quantitative models [35].

These studies have shown that wildlife remains an important food resource for the subsistence of many rural people across the region, particularly those living in small, isolated and impoverished villages nearby extensive forested areas. A number of dedicated hunters usually are present in rural communities and search for game in a selective way towards highly regarded species such as deer, pacas and large birds. These dedicated hunters sometimes manage particular habitat types (e.g. they keep a harvest portion for wildlife consumption) to attract their prey and increase their hunting success. However, most subsistence hunters frequently take their prey with very little or no management strategies in an opportunistic way while traveling to their croplands and grazing areas [7,8,24,28]. In addition to harvesting wild meat, subsistence hunting is practiced to prevent or mitigate crop damage by game species [22,37-39]. Thus, a high proportion of hunting is focused on relatively abundant and generalist species (e.g., doves, armadillos, coatis, collared peccaries, and white-tailed deer) in managed habitat types such as agricultural areas, surrounding fallows, gardens, and forest patches. However, large and threatened game species (e.g., ocellated turkey, curassows, white-lipped peccaries, brocket deer, and tapirs), which often are preferred over smaller prey, are hunted farther in mature forests, frequently without restrictions other than the hunter's skills, weapons, and time available [37,40]. Hunting for subsistence without an official permit is illegal but widely tolerated by authorities in rural Mexico [41]. The risk of not evaluating the magnitude and effects of this practice might be the loss of valuable dietary resources for many households in poverty condition [42].

Profound changes in the lifestyle of villagers are currently occurring in rural areas throughout southern Mexico and Central America. As national and local governments expand development programs introducing new roads, electricity, computers, educational and health services, and subsidies for agriculture, it is likely that people will rely less on wild resources and their traditional practices such as subsistence hunting as well as their interest in conserving game species may decrease or even disappear in many localities. In this sense, it is very important to improve and update our understanding about the current practices of wildlife use among indigenous and mestizo residents in order to support better management strategies for conserving game species and their habitat based on both scientific and traditional knowledge. Our aim in this study was to document and compare wildlife uses and hunting patterns in four rural communities near two large protected areas in the Yucatan Peninsula, southeast Mexico. Our primary questions addressed were: 1) Is wildlife still an important natural resource for rural residents of the Yucatan Peninsula?; 2) Are local hunting practices related to specific environmental, geographical, and cultural factors?; and 3) Are there wildlife management practices based on traditional ecological knowledge in the study area?

## Methods

### Study area

**Figure 1:**
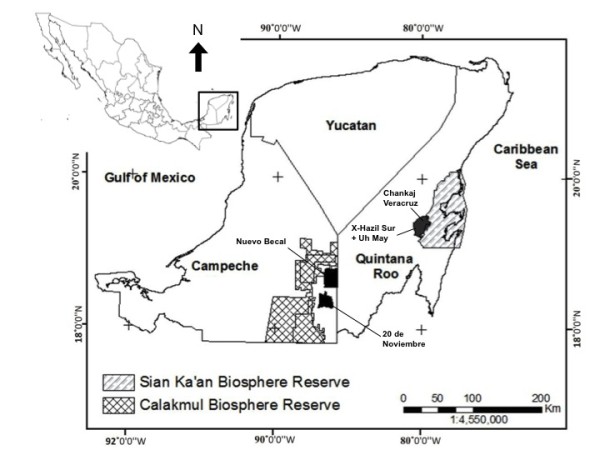
**Rural communities selected for study in the Yucatan Peninsula, southeast Mexico. **Cartographic design by David Uribe Villavicencio.

Four rural communities in the Yucatan Peninsula were selected for this study: Nuevo Becal and 20 de Noviembre nearby Calakmul Biosphere Reserve (7,238 km^2^), Campeche, and X-Hazil/Uh May and Chankaj Veracruz in the vicinities of Sian Ka’an Biosphere Reserve (6,522 km^2^), Quintana Roo (Figure 1). All these communities still contain and are surrounded by extensive tracts of evergreen and subdeciduous tropical forest interspersed with secondary vegetation, croplands and some induced grasslands for livestock production. The region has a warm and subhumid climate with most rains (600–1200 mm) falling between May and October. Tropical storms and hurricanes are relatively frequent particularly along the coast of Quintana Roo. Mean annual temperature is 26°C [43]. Limestone soils predominate in the landscape of the Yucatan Peninsula, which limits the occurrence of surface water bodies and agriculture [32].

Nuevo Becal (NB; 530 km^2^; pop = 420; [44]) is one of the largest and oldest rural settlements around Calakmul Biosphere Reserve [45]. This community was founded in the decade of 1970 by immigrants from Campeche, Veracruz, Tabasco, Chiapas, and Guatemala. Differently, most of the inhabitants of 20 de Noviembre (20NOV; 350 km^2^; pop = 350; [44]), are of Maya descent and its founders were originally from northern Campeche [46]. Most residents of both communities are farmers harvesting corn, beans, and squash for their own subsistence. Chili peppers and black pepper are commercial crops in the area. Other economic activities are the extraction of honey, timber, charcoal, gum (*Manilkara zapota*), mahogany (*Swietenia microphylla*) seeds, and palm (*Chamaedora* spp.) leaves, as well as livestock production. A few residents provide transportation services (taxis) or own small grocery stores. Hunting and fishing are traditional activities mainly for subsistence.

The communities of X-Hazil Sur and Uh May (XHZ + UHM; pop = 1,902), and Chankaj Veracruz (CHV; pop = 416; [44]), both adjacent to Sian Ka'an Biosphere Reserve, officially comprise a single territorial entity of about 550 km^2^, which is called *X-Hazil Sur y Anexos*. These two communities share close similarities in their ethnic and religious background, their settlement process, and their land use patterns. All resident families call themselves *Mayas macehuales* and descend from Maya rebels expelled from the northern Yucatan Peninsula after the Caste War in the 19th century [47]. Yucatec Maya is the predominant language, although everybody speaks Spanish with outsiders. The primary economic activities are subsistence agriculture (corn, beans, and squash), commercial crops (vegetables and chili peppers), timber extraction, apiculture, and services (small stores, public transport, and labor in the construction industry of the Riviera Maya coastal tourism region). Hunting and fishing are culturally relevant activities in these communities as well [48].

The four communities described were selected for this study based on: a) the existence of traditional uses of wildlife by their inhabitants [7,8,23,49,50]; b) their low human density; and c) their extensive forested territories in relatively good condition. We considered these attributes appropriate to test our research questions.

### Data collection and analyses

Fieldwork was done between March 2010 and March 2011. One of the authors (DSF) alternatively stayed most of the time in the four communities selected for study in order to keep records of as many hunting activities as possible. Those stays were fundamental for gaining confidence from local hunters and their families to get information about their practices related to wildlife use and management. Information was obtained through open conversations, structured and in-depth interviews, and participant observation going with hunters to search for prey (22 trips), and doing other activities along with them whenever possible. Key hunters and local assistants of each community who accepted to participate in the study were trained to properly use paper forms to keep records of hunting events. Hunting activities were recorded during a total of 534 days in the four communities combined (20NOV: 165; NB: 131; XHZ + UHM:158; CHV: 80). Nine hundred and sixty-eight forms containing records from 154 hunters were filled out by themselves, local assistants, or the researcher. Fifty-two of those hunters agreed to be interviewed (Table 1).

**Table 1:** Human population size, number of recorded and interviewed hunters, and catchment areas for hunting in four communities of the Yucatan Peninsula, southeast Mexico (March 2010 – March 2011)

	**Community**			
	**20NOV**	**NB**	**XHZ + UHM**	**CHV**
Ethnic group	Maya	Mestizo	Maya	Maya
Population size	350	420	1902	416
Hunters ^**a**^	30	37	64	23
Total interviewed	25	32	44	26
Structured interviews (hunters)	10	15	16	11
In-depth interviews (hunters and others)	11	13	20	9
Community total area (km^2^)	350	530	550 ^**b**^	550 ^**b**^
Hunting catchment area (km^2^)	240	400	455 ^**c**^	125 ^**c**^

^**a**^ Numbers of hunters whose activities were recorded in hunting forms.

^**b**^ Area shared by the two communities.

^**c**^ Areas overlapped in about 50 km^2^.

20NOV: 20 de Noviembre; NB: Nuevo Becal; XHZ + UHM: X-Hazil Sur + Uh May; CHV: Chankaj Veracruz.

The shapes of catchment areas were not circular and villages were located close to one of the community boundaries.

Species, sex, weight, date, habitat type, distance of hunting sites to the village, time elapsed, methods and instruments used were recorded for each specimen killed (see Additional file 1: Appendix 1). Record forms allowed to estimate the number of individuals of each species hunted in successful hunting trips (cited as *hunting frequency* thereafter), biomass extracted (estimated from the average weight of each species in a community), distance intervals to hunting sites, habitat types and seasonality (Dry season: November-April; Rainy season: May-October) of wildlife harvests. A few forms (N = 22) with incomplete or inconsistent recorded data were discarded from analyses. A *hunting event* was defined as a trip in which a hunter or group of hunters succeeded in capturing or killing at least one terrestrial vertebrate. Thus, unsuccessful trips were not considered in our analyses.

The catchment area within the territory of each community was assessed in square kilometers from: a) distances between hunting areas and the hunters’ houses; and b) information taken from interviews and participant hunting trips in each community. The hunters of the study area have an excellent ability to estimate distances in kilometers since most of them own motorcycles or trucks on which they use to keep records of how far from home are their croplands, ponds, preferred hunting sites, community boundaries, and other reference points. Once each reference point used for estimating distances to hunting sites was verified in a map with the help of the hunter, its coordinates were obtained with a hand held GPS. As many as possible hunting locations (roughly 68%) cited by hunters were recorded and placed on digital maps [45,46,48] to estimate catchment areas within each community using a geographic information system (ArcView 3.2; [51]) (Table 1). Harvest rates were assessed using the information on annual numbers of animals or biomass extracted per unit area (km^2^) in each community.

Identification of game species was facilitated by the experience of two of the authors (EJN, JLR) working over two decades with wildlife in southeast Mexico. Photographs previously taken by the authors and color plates from field guides [52,53] were helpful in corroborating the identity of mammals and birds hunted and cited using local names during the interviews. Only mammals, birds, and reptiles heavier than 0.5 kg were considered for hunting records in this study, basically because residents minimized the importance of capturing small species such as squirrels, quails, doves, and small parrots for any purpose. Consequently, local hunters did not consider worthwhile to mention such small species during the interviews neither to be included in the record forms because their capture was more a hobby for kids and teenagers rather than for family subsistence.

Data were analyzed with parametric and non-parametric tests of one-way Analysis of Variance (ANOVA), and Generalized Lineal Models (GLM). Prior to analyses, variables were checked for normality with Shapiro-Wilks W test and for equality of variance with Bartlett’s test [54]. When data did not meet the parametric assumptions, nonparametric Wilcoxon/Kruskal-Wallis H rank tests were used. If differences were significant, a post-hoc Tukey-Kramer test was performed to identify variation between pairs of means. Regression analyses were also performed to examine the explanatory variation of number of animals hunted by distance intervals (0–5 km; 5–10 km; 10–15 km; 15–20 km; 20–25 km; 25–30 km; and >30 km). *Poisson* Generalized Linear Mixed Model (PGLMM) was used to test the effect of explanatory variables such as species and communities, and also to test one-way interaction terms between those explanatory variables or source of variation [55,56]. Statistical analyses were performed with JMP 7 (The SAS Institute, Inc. 2007). All means are presented ± 1 SE and considered marginally significant if 0.10 < P > 0.05, and significant at P ≤ 0.05.

The Division of Graduate Studies and the Ethics Committee of El Colegio de la Frontera Sur approved the research project “Subsistence hunting, management and conservation of wildlife in rural communities of the Yucatan Peninsula, México” to Didac Santos-Fita on September 4, 2009. The methods used in this project are in compliance with the Helsinki Declaration.

## Results

### General description of hunting and uses of species

We recorded nine different uses on 25 body parts of 46 species (26 mammals, 16 birds, and 4 reptiles; Table 2). Most purposes of use (obtaining food, medicinal products, tools, adornments, pets, mitigating damage, and sale) and body parts of animals hunted were similar among the four communities. Ritual uses (meat in religious ceremonies) were only observed in small groups of Maya hunters in XHZ + UHM and CHV. We found isolated cases of wildlife husbandry in 20NOV (Pond slider turtles, *Trachemys scripta*), and in CHV (collared peccaries, *Pecari tajacu*).

**Table 2:** Terrestrial vertebrates used by residents of the study area

**Species**	**English name**	**Maya name**	**Record type**	**USE [part used] by community**							
				**20NOV**		**NB**		**HXZ + UHM**		**CHV**	
MAMMALS											
*Odocoileus virginianus*	White-tailed deer	Kéej	H	F	[m]	F	[m]	F	[m]	F	[m]
				MD	[f, h]	MD	[f]	MD	[f]	MD	[f]
				T	[ho]	T	[bo]	T	[bo, t, sk]	T	[bo, ho]
				AD	[ho, h, sk]	AD	[ho, sk]	AD	[ho]	AD	[ho]
								C	[m]	C	[m]
*Mazama americana; M. pandora*	Red brocket deer	Yuk	H	F	[m]	F	[m]	F	[m]	F	[m]
				MD	[f, h]	MD	[f]	MD	[f]	MD	[f]
				AD	[ho, h, sk]	T	[bo]	T	[bo, ho, t]	AD	[ho]
						AD	[ho]	AD	[ho]	C	[m]
								C	[m]		
*Pecari tajacu*	Collared peccary	Kitam	H	F	[m]	F	[m]	F	[m]	F	[m]
				MD	[h, sg]	MD	[sg, l]	MD	[h]	MD	[h]
				AD	[ja]	T	[bo]	T	[ja]	C	[m]
				DC		DC		C	[m]	DC	
								DC		H	
								H			
*Tayassu pecari*	White-lipped peccary	K'áaxil k'éek'en	H; Re	F	[m]	F	[m]	F	[m]	F	[m]
*Dasyprocta punctata*	Central American agouti	Tsuub	H; Re	F	[m]	F	[m]	F	[m]	F	[m]
			Ob	MD	[ha]			MD	[ha, bo]		
				P							
*Cuniculus paca*	Paca	Jaaleb	H	F	[m]	F	[m]	F	[m]	F	[m]
						MD	[bo, bi]	MD	[bi]	P	
								AD	[s]	H	
								P			
								H			
								C	[m]		
*Dasypus novemcinctus*	Nine-banded armadillo	Weech	H; Re	MD	[b, sh]	F	[m]	----	----	----	----
						MD	[b, t]				
*Nasua narica*	White-nosed coati	Chi’ik	H	F	[m]	F	[m]	F	[m]	F	[m]
				MD	[g, f]	MD	[m, g]	MD	[g]	DC	
				DC		DC		DC			
*Tapirus bairdii*	Baird’s tapir	Tsíimin	H; Re	DC		DC		DC		DC	
						F	[m]	F	[m]		
						MD	[h]				
*Orthogeomys hispidus*	Hispid pocket gopher	Baj	H	DC		F	[wb]	F	[wb, ex]	F	[wb, ex]
				F	[wb]	DC		MD	[ja]	DC	
				MD	[bo]			DC			
*Panthera onca*	Jaguar	Báalam; Chak mo’ol	H; Re	MD	[f]	DC		MD	[f]	MD	[f]
				AD	[fa]	AD	[fa]	AD	[fa]	AD	[fa]
				DC		S	[fa, sk]	S	[fa]	S	[fa]
						MD	[f]	DC		DC	
*Puma concolor*	Cougar	Coj	H; Re	MD	[f]	DC		MD	[f]	MD	[f]
				AD	[fa]	AD	[fa]	AD	[fa]	AD	[fa]
				DC		S	[fa]	S	[fa]	S	[fa]
								F	[m]	F	[m]
								DC		DC	
*Leopardus pardalis*	Ocelot	Sak xikin	Re	¿?	¿?	P		AD	[fa, wb]	AD	[fa]
*Leopardus wiedii*	Margay	¿?	Re	P		DC		MD	[m]	----	----
*Herpailurus yagouaroundi*	Yaguarundi	“Ca” coj	H; Re	¿?	¿?	¿?	¿?	AD	[fa]	AD	[fa]
*Ateles geoffroyi*	Geoffroy’s spider monkey	Ma’ax	Ob	P		----	----	P		MD	[m, f]
								MD	[c, gr]		
*Alouatta pigra*	Black howler monkey	Ba’ats’	Re	----	----	----	----	MD	[m, f]	MD	[m, f]
*Procyon lotor*	Northern raccon	K’ulu	Re	DC		DC		DC		DC	
*Potos flavus*	Kinkajou	Áak’ab ma’ax	Ob	----	----	F	[m]	----	----	----	----
						AD	[sk]				
*Eira barbara*	Tayra	San jo’ol	H	----	----	----	----	DC		DC	
*Didelphis marsupialis*	Common opossum	Ooch	Re	----	----	----	----	MD	[m]	----	----
*Conepatus* sp*.*	Skunk	Pay ooch	Re	MD	[m, wb]	MD	[m, f]	----	----	----	----
*Coendu mexicanus*	Mexican porcupine	K’ixpachoch	Re	----	----	MD	[sp]	MD	[sp]	----	----
*Tamandua mexicana*	Northern tamandua	Chaab	Re	----	----	MD	[m]	----	----	----	----
*Sciurus* sp.	Squirrel	Ku’uk	Ob	----	----	----	----	P		----	----
								F	[m]		
Rodentia	Mice	¿?	Re	----	----	----	----	MD	[ex]	MD	[ex]
BIRDS											
*Ortalis vetula*	Plain Chachalaca	Baach	H; Re	F	[m]	F	[m]	F	[m]	F	[m]
				P							
*Penelope purpurascens*	Crested Guan	Koox	H; Re	F	[m]	F	[m]	F	[m]	F	[m]
*Crax rubra*	Great Curassow	K’anbul	H	F	[m]	F	[m]	F	[m]	F	[m]
				MD	[f]			MD	[cr]	T	[fe]
*Meleagris ocellata*	Ocellated Turkey	Kúuts	H	F	[m]	F	[m]	F	[m]	F	[m]
				MD	[f]	AD	[fe]	AD	[sr]	AD	[sr]
				T	[sr]			T	sr, fe]	S	[sr]
				S	[sr]			S	[sr]		
*Cryptullerus* sp.	Tinamou	¿?	H; Re	F	[m]	F	[m]	----	----	----	----
Columbidae	Pigeon	¿?	Ob	P		----	----	P		----	----
*Tinamus major*	Great Tinamou	Noom	Re	----	----	----	----	F	[m]	F	[m]
*Colinus virginianus*	Northern bobwhite	Beech’	Ob	----	----	----	----	P		----	----
								F	[m]		
Trochilidae	Hummingbird	Ts’unu’un	Re	----	----	----	----	MD	[b]	----	----
*Ramphastos sulfuratus*	Keel-billed Toucan	Pan ch’eel	Ob	----	----	----	----	----	----	P	
*Sarcoramphus papa*	King Vulture	Ch’oom	Re	----	----	----	----	----	----	MD	[wb]
*Coragyps atratus*	Black Vulture	Ch’oom	Re	----	----	MD	[m, fe]	----	----	MD	[fe]
*Amazona* sp.	Parrot	T’uut’	Ob	P		P		P		P	
				DC		DC		F	[m]	DC	
								DC			
Picidae	Woodpecker	¿?	Re	----	----	MD	[he]	----	----	----	----
*Buteogallus* sp*.*	Black Hawk	¿?	Re	----	----	DC		----	----	----	----
*Buteo* sp	Hawk	¿?	Re	----	----	DC		----	----	----	----
REPTILES											
*Crocodylus moreletii*	Morelet’s crocodile	¿?	Ob	P		----	----	----	----	----	----
Kinosternon sp.	Mud turtle	¿?	Ob; Re	H		MD	[b, m]	MD	[b]	MD	[b]
*Trachemys scripta*	Red-eared slider	Ka’a nix	H; Re;	F	[m]	F	[m]	F	[m]	F	[m]
			Ob	MD	[b]	MD	[b]				
				H							
*Crotalus durissus*	Tropical rattlesnake	Ts’aab kaan	Re	MD	[m, f, r]	MD	[m, f, r]	MD	[m, f, wb]	MD	[m, f, wb, mo]

H = hunted; Ob = observed; Re = reported.

**Purpose of use**: F = food; MD = medicine; T = tool; AD = adornment; P = pet; DC = damage control; H = husbandry; C = ceremonial/religious; S = sale.

**Part used**: m = meat; b = blood; bo = bone; s = skull; ja = jaw; sk = skin; ha = hair; f = fat/oil; l = liver; bi = bile; h = hoof; g = genital; t = tail; sp = spine; ho = horn/antler; cr = crest/crown; sh = shell; he = heart; fe = feather; sr = spur; fa = fang; sg = scent gland; ex = excrement; mo = moult; r = rattle; wb = whole body.

Species identified with field guides for mammals (Reid 2009) and birds (Howell and Webb 1995).

20NOV: 20 de Noviembre; NB: Nuevo Becal; XHZ + UHM: X-Hazil Sur + Uh May; CHV: Chankaj Veracruz.

Data recorded between March 2010 and March 2011.

During our study, only one man (in 20NOV) out of 153 persons (about 5% of total population) detected as wildlife users in the four communities visited, could be regarded as a “full-time hunter”. This man hunted year-round one or more animals almost every day either to feed his family or selling some meat in his community, extracting by himself about 30% of total prey taken there, and roughly 10% of all prey in the four communities combined. According to the 52 hunters interviewed in this study, hunting is practiced primarily for obtaining food (80%; including occasional sale), mitigating crop damage (10%), recreation (6%), and getting money from prey sales (4%). However, virtually all hunters harvest animals with two or more purposes in mind (e.g., 12 species were hunted for their meat and medicinal byproducts, using 13 different body parts).

Hunters interviewed in 20NOV and NB considered that unlike past decades, they now have a wider array of productive alternatives (e.g., new commercial crops, timber, honey, chicle, and charcoal production) discouraging frequent hunting for subsistence or trade. Stronger law enforcement by federal authorities and military check points along primary roads have also been deterrents to frequent hunting in the communities visited. Consequently, most hunters currently refrain from killing animals beyond their own lands and for purposes other than providing meat for their families. In fact, roughly 65% of local hunters practiced this activity only sporadically, particularly during their trips to their working plots. These “sporadic” hunters went out alone or in small groups and took prey when the opportunity arose. They rarely sold or traded their killings.

Diurnal active search was the most frequent hunting technique used in 20NOV and NB, followed by nocturnal waiting on trees using flashlights for shooting pacas (*Cuniculus paca*). Hunters of NB took advantage of locally trained dogs to find pacas and armadillos (*Dasypus novemcinctus*) in burrows. In 20NOV there are occasional group hunting trips with dogs, called *batidas.* These hunting groups of up to 10 people organize a noisy drive of potential prey that are killed at a strategic point by waiting shooters along pathways used by animals [10,57]. Unfortunately, some local young men in this community are adopting a new hunting style on four-wheeled all-terrain vehicles just for fun, having no need for food or money from wildlife products.

Residents from XHZ + UHM and CHV communities said that hurricane *Dean* (2007) severely affected hunting by causing high mortality rates, starvation and migration in many game populations. In addition, access roads and pathways to hunting sites were blocked by fallen trees. Hunters in XHZ + UHM conceded that some valuable wildlife populations had recovered after the hurricane and could be used again. Large birds such as the Great Curassow (*Crax rubra*), Ocellated Turkey (*Meleagris ocellata*) and Crested Guan (*Penelope purpurascens*) were not part of these recovered species and their harvest rates remained low in the area. Contrastingly, pacas seemed to be benefited after the hurricane hit, which favored their shelter availability and protected them from hunters for about three years. This is likely one of the reasons why the paca is the most frequent killed mammal in the area. Hunters of XHZ + UHM mentioned that after hurricane *Dean*, waiting on trees (*espiar*) in the forest for a variety of species (not only paca) has become a more frequent practice than active search form them.

Hunting in XHZ + UHM is both for family consumption and occasional sale within the community and beyond. We had evidence of three hunters that regularly provide wild meat to selected buyers in Felipe Carrillo Puerto (pop = 25,000; [44]), a small city 30 km away by a paved road without military check points. Besides solitary hunting, small group hunting is frequent in XHZ + UHM. Members of these groups organized *batidas* without dogs and shared their prey evenly. These hunters stated that 10 years ago they stopped doing *batidas* for ceremonial and religious purposes related to agricultural cycles.

Hunting in CHV is a typically individual activity usually practiced for obtaining meat. However, its proximity to Felipe Carrillo Puerto (just 12 km away) was an opportunity for a few hunters to get higher prices for their prey. *Batidas* were frequently used in the past for rituals associated to agricultural cycles. Nevertheless, they are no longer organized in CHV because some hunters were accidentally shot and killed while practicing this hunting technique.

The instruments used for hunting in the four communities visited were shotguns (calibers .16, .20, and .12), .22 caliber rifles, and rudimentary traps especially for catching pocket gophers (*Orthogeomys hispidus*) and live pacas in XHZ + UHM and CHV. The principal vehicles for hunters of the four communities were bicycles, motorcycles, and horses (in 20NOV and NB). Some hunters use cars and trucks (except in CHV) for transportation, while others move by foot to their hunting sites.

### Hunting sites, hunting frequencies, and harvest rates

Hunting sites were always located within the territory of each community. The landscape of communal territories consisted primarily of habitat mosaics where croplands, fallows, grasslands, rainforest patches (both mature and successional), and seasonal wetlands and lagoons of different sizes intermix. Hunting usually occurs: 1) around water bodies and wetlands, especially during the dry season; 2) in croplands, during the harvest period between September and February; and 3) in forest patches, when hunters are not busy maintaining their crops.

A total of 968 terrestrial vertebrates of 20 species (14 mammals, 5 birds, and a reptile) were hunted or captured in 664 successful events recorded in the four communities visited during the study (Table 3). Mammals constituted the most frequently hunted group (n = 555; 57.3% of total prey). However, two birds were the most frequently hunted species: the Great Curassow (n = 235; 24.3%; which was the most hunted in three communities), and the Ocellated Turkey (n = 144; 15%). After these two species, the paca (n = 141; 14.6%), the white-tailed deer (*Odocoileus virginianus*: n = 112; 11.6%), and the collared peccary (n = 103; 10.6%) were the following species hunted with the highest frequency in the four communities. The remaining mammals (n = 11), birds (n = 3), and a reptile represented 23.9% of total prey taken by hunters. The total number of animals hunted was consistent among communities (Kruskal-Wallis’s H = 3.35; df. 3; P = 0.34). Similarly, there were no significant differences in the numbers of mammals hunted (H = 1.60; df. 3; P = 0.66) and birds hunted (H = 3.94; df. 3; P = 0.27) among communities.

**Table 3:** **Numbers of animals (N) hunted and harvest rates (HR: individuals hunted/10 km**^**2**^**/year) of wildlife species in the study area**

**Species**			**Community**						**Total**	
	**20N0V**		**NB**		**HXZ + UHM**		**CHV**			
	**N hunted**	**HR**	**N hunted**	**HR**	**N hunted**	**HR**	**N hunted**	**HR**	**N hunted**	**HR**
*Cuniculus paca*	11	0.5	16	0.4	90	2	24	2	141	4.9
*Odocoileus virginianus*	16	0.7	36	0.9	52	1.1	8	0.6	112	3.3
*Pecari tajacu*	16	0.7	31	0.8	32	0.7	24	2	103	4.2
*Dasyprocta punctata*	46	1.9	2	0.1	8	0.2	0	0	56	2.2
*Orthogeomys hispidus*	2	0.1	2	0.1	32	0.7	12	1	48	1.9
*Mazama pandora; M. americana*	23	1	11	0.3	1	0.02	8	0.6	43	1.9
*Nasua narica*	4	0.2	2	0.1	13	0.3	3	0.2	22	0.8
*Tayassu pecari*	2	0.1	7	0.2	0	0	2	0.2	11	0.5
*Dasypus novemcinctus*	0	0	9	0.2	0	0	0	0	9	0.2
*Puma concolor*	0	0	0	0	2	0.04	1	0.1	3	0.1
*Panthera onca*	0	0	2	0.1	1	0.02	0	0	3	0.1
*Tapirus bairdii*	0	0	1	0.02	1	0.02	0	0	2	0.04
*Herpailurus yagouaroundi*	0	0	0	0	1	0.02	0	0	1	0.02
*Eira barbara*	0	0	0	0	1	0.02	0	0	1	0.02
TOTAL MAMMALS	120	5.2	119	3.2	234	5.1	82	6.7	555	20.2
*Crax rubra*	115	4.8	68	1.7	24	0.5	28	2.2	235	9.2
*Meleagris ocellata*	64	2.7	52	1.3	22	0.5	6	0.5	144	5
*Penelope purpurascens*	10	0.4	2	0.1	1	0.02	0	0	13	0.52
*Ortalis vetula*	3	0.1	4	0.1	2	0.04	0	0	9	0.24
*Crypturellus* sp.	2	0.1	1	0.02	0	0	0	0	3	0.12
TOTAL BIRDS	194	8.1	127	3.2	49	1.1	34	2.7	404	15.1
*Trachemys scripta*	1	0.04	0	0	8	0.2	0	0	9	0.2
TOTAL REPTILES	1	0.04	0	0	8	0.2	0	0	9	0.2
OVERALL	315	13.3	246	6.4	291	6.4	116	9.3	968	35.5

20NOV: 20 de Noviembre, catchment area (ca) = 240 km^2^; NB: Nuevo Becal, ca = 400 km^2^; XHZ + UHM: X-Hazil Sur + Uh May, ca = 455 km^2^;

CHV: Chankaj Veracruz, ca = 125 km^2^.

Data recorded between March 2010 and March 2011 (12 months in each community).

The last column shows the sums of animals hunted and harvest rates of the four communities surveyed.

In addition to the five species mentioned above, pocket gophers (n = 48; 5%), brocket deer (*Mazama* sp.: n = 43; 4.4%), and coatis (*Nasua narica*: n = 22; 2.3%), were taken by hunters of the four communities. The number of animals hunted within the eight species did not vary across communities (PGLMM, X^2^ = 2.76; df. 3; P = 0.43). While the number of animals hunted that occur in the four communities vary highly among the eight species (X^2^ = 69.22; df. 7; P < 0.001). However, the variation of the number of animals hunted across species was highly dependent on the community (Species*Community interaction term; X^2^ = 94.45; df. 21; P < 0.001). The Great Curassow and the Ocellated Turkey were more frequently hunted than the other six species (mammals) in 20NOV and NB**.** In XHZ + UHM, pacas and white tailed-deer were the most frequently hunted species, while Curassows and Ocellated Turkeys were 5–6 times less hunted than pacas (see Table 3).

**Figure 2:**
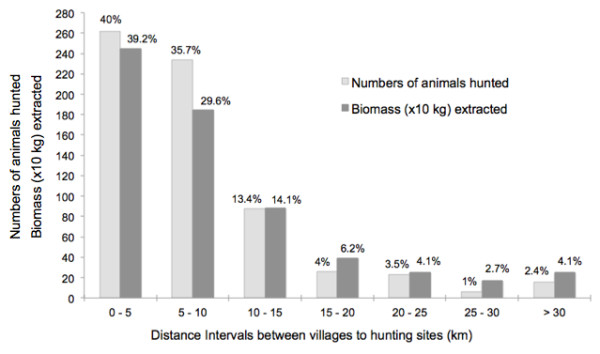
**Overall numbers of animals hunted and biomass extracted (x10 kg) by distance interval in four communities of the Yucatan Peninsula, southeast Mexico. **Biomass extracted was estimated using average weights of each species in each community. Biomass data shown in the graph correspond to the hunting records for which distances were available (N = 655; 67.7%). Data recorded between March 2010 and March 2011.

Overall numbers of animals hunted and biomass extracted declined as hunting distances increased from villages. The data distribution of animals hunted and biomass extracted responded to a Weibull (*Poisson* type) distribution (Cramer-von Mises W Test ≥0.07, P ≤ 0.10; Figure 2). Within the eight species previously mentioned as hunted in all communities (Great Curassow, Ocellated Turkey, paca, white-tailed deer, collared peccary, pocket gopher, brocket deer, and coati), the distance intervals where most preys were caught from each community were 5–10 km in 20NOV (r = −1.56; F_2, 53_ = 8.05; P < 0.001), 0–10 km in NB (r = −1.22; F_2, 53_ = 15.47; P < 0.001), and 0–5 km in HXZ + UHM (r = −1.30; F_2, 53_ = 16.83; P < 0.001) and CHV (r = −0.99; F_2, 53_ = 17.50; P < 0.001). Interestingly, compared to intermediate distances (10–25 km), a slight increase in animals hunted was detected at distances over 30 km from the villages. Hunting frequencies were similar in forested and agricultural areas for all communities combined (H = 0.58; df. 1; P = 0.45).

**Figure 3:**
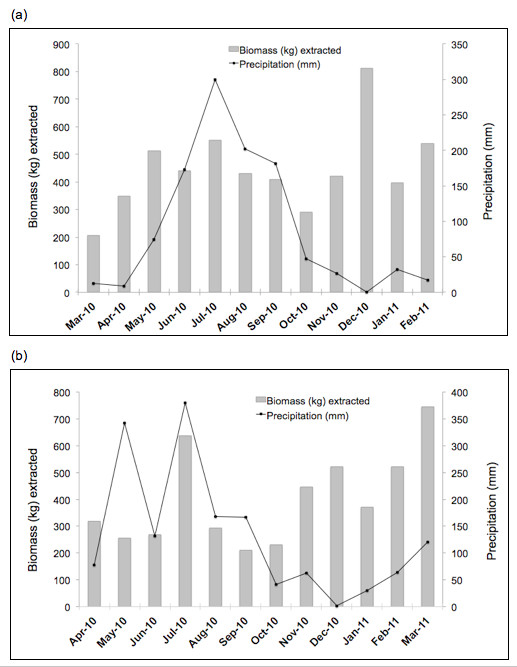
**Monthly wildlife biomass (kg) extracted and precipitation in communities of the Yucatan Peninsula, southeast Mexico: (a) 20 de Noviembre and Nuevo Becal, Calakmul area; and (b) X-Hazil Sur + Uh May and Chankaj Veracruz, Sian Ka’an area. **Dry season: November-April; Rainy season: May-October. Data are shown in two graphs due to different precipitation and fieldwork periods in each area: **(a) **Calakmul area: March 2010 – February 2011; and **(b) **Sian Ka’an area: April 2010 – March 2011.

Estimated annual biomass extracted was 10,190 kg, of which mammals (N = 555) represented 83.9%, birds (N = 404) were 15.7%, and reptiles (N = 9) only 0.4% (Table 4). The most frequently hunted species were also the ones providing the highest biomass volumes for users: white-tailed deer (3,692 kg; 36.2%), collared peccary (2,158 kg; 21.8%), Great Curassow (938 kg; 9.2%), paca (824 kg; 8.1%), and Ocellated Turkey (614 kg; 6.0%). These five species comprised 8,226 kg or 80.7% of total annual biomass and their pooled harvest rate was 276 kg/10 km^2^/year, versus only 1,964 kg and 70.5 kg/10 km^2^/year for the remaining 15 species. Mean annual biomass extracted by hunter was 67 ± 13.5 (SD) kg, while annual *per capita* consumption was 4.65 ± 2.7 (SD) kg. Monthly biomass extracted during the dry / rainy season were 2,722.4 kg / 2,633.6 kg in the Calakmul (20NOV + NB communities) area, and 2,926.1 kg / 1,907.9 kg in the Sian Ka’an (XHZ + UHM + CHV communities) area (Figure 3). About 63.3% of animals hunted in the two study areas (all communities) combined were recorded during the dry season, which corresponded to 55.4% of total biomass extracted. December was a remarkable month for hunting and biomass extracted (812.1 kg) in Calakmul area, where 117 individuals (mostly great curassows) were killed. Finally, harvest rates estimated for all 20 species hunted in all communities combined were 35.5 individuals/10 km^2^/year and 347 kg/10 km^2^/year (Tables 3 and 4). In spite that catchment areas in NB (400 km^2^) and XHZ + UHM (455 km^2^) are considerably larger than in 20NOV (240 km^2^) and CHV (125 km^2^), biomass harvest rates (80–100 kg/10 km^2^/year), were similar across communities.

**Table 4:** **Annual biomass extracted (BE: kg) and harvest rates (HR: kg/10 km**^**2**^**/year) of wildlife species in the study area**

**Species**			**Community**						**Total**	
	**20N0V**		**NB**		**HXZ + UHM**		**CHV**			
	**BE**	**HR**	**BE**	**HR**	**BE**	**HR**	**BE**	**HR**	**BE**	**HR**
*Cuniculus paca*	62	2.6	99.1	2.5	524.5	11.5	138.7	11.1	824	27.7
*Odocoileus virginianus*	522.2	21.8	1,144.5	28.6	1,854.2	40.7	171.2	13.7	3,692	104.8
*Pecari tajacu*	303.4	12.6	727.1	18.2	592	13	535.5	42.8	2,158	86.6
*Dasyprocta punctata*	137.9	5.7	7	0.2	21.3	0.5	0	0	166	6.4
*Orthogeomys hispidus*	1	0.04	1	0.02	16	0.4	6	0.5	24	0.96
*Mazama pandora; M. americana*	322	13.4	193.8	4.8	15	0.3	133.3	10.7	664	29.2
*Nasua narica*	16.9	0.7	10	0.2	44.9	1	11.3	0.9	83.5	2.8
*Tayassu pecari*	60	2.5	140	3.5	0	0	60	4.8	260	10.8
*Dasypus novemcinctus*	0	0	36	0.9	0	0	0	0	36	0.9
*Puma concolor*	0	0	0	0	60	1.3	30	2.4	90	3.7
*Panthera onca*	0	0	90	2.2	45	1	0	0	135	3.2
*Tapirus bairdii*	0	0	200	5	200	4.4	0	0	400	9.4
*Herpailurus yagouaroundi*	0	0	0	0	7.5	0.2	0	0	7.5	0.2
*Eira barbara*	0	0	0	0	5	0.1	0	0	5	0.1
TOTAL MAMMALS	1,425.4	59.34	2,648.5	66.21	3,385.4	74.4	1,086	86.9	8,545	286.8
*Crax rubra*	448	18.6	275.9	6.9	103.3	2.3	110.4	8.8	938	36.7
*Meleagris ocellata*	256	10.7	257.1	6.4	83.3	1.8	17.5	1.4	614	20.3
*Penelope purpurascens*	24.4	1	5	0.1	2.5	0.05	0	0	32	1.2
*Ortalis vetula*	4.5	0.2	6	0.2	3	0.1	0	0	13	0.4
*Crypturellus* sp.	2	0.1	1	0.02	0	0	0	0	3	0.1
TOTAL BIRDS	734.9	30.6	545	13.6	192.1	4.2	127.9	10.2	1,600	58.7
*Trachemys scripta*	5	0.2	0	0	40	0.9	0	0	45	1.1
TOTAL REPTILES	5	0.2	0	0	40	0.9	0	0	45	1.1
OVERALL	2,165.3	90.14	3,193.5	79.81	3,617.5	79.5	1,213.7	97.1	10,190	347

20NOV: 20 de Noviembre, catchment area (ca) = 240 km^2^; NB: Nuevo Becal, ca = 400 km^2^; XHZ + UHM: X-Hazil Sur + Uh May, ca = 455 km^2^;

CHV: Chankaj Veracruz, ca = 125 km^2^.

Data recorded between March 2010 and March 2011 (12 months in each community).

The last column shows the sums of animals hunted and harvest rates of the four communities surveyed.

## Discussion

### General description of hunting and uses of species

Hunters and other rural inhabitants of southeast Mexico have good knowledge on biological and behavioral aspects of wildlife in their communities, which often leads to specific hunting purposes, strategies, and techniques. In general terms, wildlife use, hunting patterns, and technologies observed in the four communities visited in this study are similar to those recorded in previous studies for rural Mayan and mestizo communities in the Yucatan Peninsula [10,24-26,58]. Unlike other study areas where indigenous groups hunted a wider array of game species than non-indigenous residents [18,28], we did not find significant differences in the patterns of wildlife use between Mestizo hunters of NB, and Maya hunters of the other three communities visited. The most heavily hunted species in the four communities were those providing more products and by-products for residents, such as the Great Curassow, Ocellated Turkey (endemic of the Yucatan Peninsula; [59]), deer, peccaries, paca, agouti, and armadillos. Most of these species are also frequently hunted in other Neotropical sites [3,6,18,28,60].

All hunting techniques documented in our study sites (active search; nocturnal waiting on trees using flashlights, opportunistic hunting; and *batidas*) are traditionally practiced in southeast Mexico [7,8,26,61]. Hunting techniques in the Yucatan Peninsula usually depend on biological and behavioral features of the target species. A good example of this was found for the paca, a nocturnal and solitary species mostly hunted by silent waiting on trees using flashlights. Interestingly, one of the oldest and most cooperative technique (the *batida*), remains frequent in the northern Yucatan State, where high human densities and forest fragments prevail [10,25,26,58], while it is rare in the southern Yucatan Peninsula, where extensive forests remain around small villages. Similarly, ancient weapons (e.g., spears, bows and arrows) as well as rustic traps are no longer used in our study area, while firearms are widespread.

Although high numbers of potential game species were available around each community in our study area, local hunters targeted just a few of them. This is consistent with findings of previous studies in South America [3,62-64], and southeast Mexico [7,8,23,24,61], where most contemporary hunters no longer rely on wild meat as their primary source of animal protein and concentrate in relatively small sets of large and medium-sized species. In fact, hunters of the Yucatan Peninsula typically take between 12 and 25 species (mostly mammals and birds), compared to 32 species hunted in the Lacandon Forest of Chiapas [28], and 40–60 species (considering prey under 0.5 kg) taken by indigenous groups of South America such as the Yuquí in Bolivia [65], the Siona-Secoya in Ecuador [66], the Aché in Paraguay [64], and the Huaorani in Ecuador [67,68].

Ecological (e.g., carrying capacity, habitat degradation or fragmentation), cultural (e.g., dietary taboos), and socio-economic factors (e.g., income, employment, and availability of domestic meat) may explain spatiotemporal variations in local hunting patterns and preferences [62,69]. In the Yucatan Peninsula, hunting persists as a cultural practice. However, very few residents may be regarded as full-time hunters, and subsistence hunting is no longer a primary economic activity. Local hunters currently complement their diet by purchasing meat and raising livestock and poultry. In this sense, it is likely that rural inhabitants of our study area tend to depend less on hunting for subsistence as they get more access to an increasing number of productive alternatives [7,8,28]. This factor made it difficult to get more detailed information on wild and domestic meat consumption as well as on the economics of hunting at the household level in the communities selected for study. In fact, the sale of wild meat is a sensitive issue in our study area considering that residents are aware of the illegal status of this activity in addition to a generalized lack of official licenses for firearms possession. As most participant hunters avoided providing information on wild meat sales in the forms supplied and during the interviews, we decided not to exert pressure on them in order to keep their confidence in our team. This was the main reason why we focused our analyses on communities rather than in hunters’ households. It will be important to design new surveys in which a sample of households in each community is closely monitored for hunting events and animals hunted during shorter periods (e.g., three months). Those surveys should consider a stronger focus in the economics of local hunting in order to assess its impact in the livelihoods of hunters and to estimate the magnitude of wildlife trade in the Yucatan Peninsula, which is still poorly known.

### Hunting sites, hunting frequencies, and harvest rates

Although we did not estimate hunting preferences [70-73], residents of the four communities repeatedly commented on their special appreciation for medium and large-sized prey with fine meat and good taste such as deer, pacas, peccaries, turkeys, and curassows. These were actually the most frequently hunted species providing the largest portion of wildlife biomass harvested in our study area. These animals have been among the most important prey taken wherever they exist in southeast Mexico [10,23,26-28,49]. The white-tailed deer deserves a special mention because of its high nutritional and cultural importance for the Maya of the Yucatan Peninsula [25]. This ungulate is very tolerant to forest fragmentation, which has favored an increase in its distribution range and abundance as more forested areas are cleared for agriculture and cattle ranching [36,74]. These activities induce mosaics of forest fragments, croplands (mostly Mayan *milpas*, containing maize, beans, squash, and other cultivated plants), and grasslands that constitute eventual hunting sites in the Yucatan Peninsula.

An interesting case is that of the armadillos, which have been previously reported as rarely used in the Yucatan Peninsula by Maya people [7,8,23,26,50]. In our study area, these mammals are sometimes taken for medicinal purposes by Maya residents of 20NOV. Armadillos are generally rejected as food by most contemporary Peninsular Maya apparently due to their “poor taste” and the presence of undesirable fat nodules in their meat (also cited by Jorgenson [7]). Nonetheless, these animals are still very appreciated as food by the Lacandon Maya and other indigenous groups of Chiapas [27,28] and South America [65,75,76].

Hunting of primates (black howler –*Alouatta pigra*– and spider –*Ateles geoffroyi*– monkeys) has become extremely rare in Maya and Mestizo communities of southeast Mexico, probably due to cultural change in contemporary hunters [7,23,24,28]. However, monkeys were frequently used for meat by past generations of the Lacandon Maya [27,77], and they are still frequently consumed by several ethnic groups in the Amazon [5,14-16,18,67]. In the case of large predators such as jaguars, during the period of study we recorded two of them hunted by farmers in NB, who expected to mitigate predation on their livestock (over 36 cows and sheep were killed by jaguars in a year; [78]), and one more killed in XHZ + UHM because of fear of attacks on villagers and because this action is assumed appropriate by local hunters to reduce competition for prey such as pacas, peccaries, and deer.

Large birds such as the Great Curassow and the Ocellated Turkey are extremely important for Maya and mestizo hunters representing around 40% (53.3% in 20NOV and NB) of total prey taken in the communities of our study area. Previous studies in the Calakmul area [23; Calmé et al., unpublished data) had already documented frequent hunting of these two game birds. However, December 2010 was particularly productive for Great Curassow hunters of 20NOV and NB (47 and 30 birds taken, respectively). Residents of these two communities stated that curassows and turkeys were probably pressed by an unusual drought (and food scarcity) to go into the *milpas* during the harvest period, which facilitated their killing. In spite that the distribution range of the two species has been shrinking in the Yucatan Peninsula [59], their populations appear to be large enough in the Calakmul area to support a high hunting pressure in 20NOV and NB. Nevertheless, the sustainability of their hunting in the area remains to be addressed.

A different situation has been taking place for curassows and turkeys around Sian Ka’an Biosphere Reserve, where they represented 19.6% of all animals hunted in XHZ + UHM and CHV. Hurricane *Dean* (2007) severely affected hunting in this area by causing habitat destruction, high mortality rates, starvation and migration in many game populations. This was documented by Ramírez-Barajas [79], who estimated a two-third decrease in overall abundance of game species after the hit of *Dean*. Hunters of XHZ + UHM and CHV agreed that populations of large game birds were unusually low after the hurricane, which made them more difficult to find. Yet, we did not detect a shift in the hunters’ choice for large game birds over smaller species (e.g., chachalacas and tinamous) in the Sian Ka’an area.

The pattern of animals hunted and biomass extracted from all game species in the four communities showed that their harvests are concentrated within 10 km from the villages, and we did not detect differences in the numbers of animals hunted between croplands and forested areas, which in most cases were contiguous. This differs with the findings of Smith [51] in western Panama, where hunting occurs mainly in gardens and fallows near indigenous settlements. In our study locations, frequent hunting sites may be at distances over 20 km from settlements because: 1) villages are placed close to one of the boundaries of their extensive territories, which implies that catchment areas are not circular and may be in the vicinities of unpopulated forests belonging to either other communities or large protected areas (e.g., Calakmul and Sian Ka’an Biosphere Reserves); 2) water scarcity limits agriculture to relatively small plots interspersed with either fallows or mature forest patches far away from the villages; and 3) many hunters own bicycles or motorcycles to commute to their distant plots or preferred hunting sites in a relatively short time. The slight increase in animals hunted detected at distances over 30 km from the villages may be a result of the knowledge of high quality hunting and fishing sites by skilled hunters who enjoy camping in remote areas for 2–3 days with a few relatives or close friends. This was the case of several lagoons (*aguadas*) in NB and 20NOV, and an extensive savanna between XHZ + UHM/CHV and Sian Ka’an Biosphere Reserve. Considering that hunting areas increase with distance to villages, it is important to note that wildlife harvest rates may not necessarily differ between distance intervals. Although this discussion is beyond the reach of our dataset, information on hunting in relation to distance from villages may be helpful to improve habitat management practices and identify sensitive areas for community-based conservation of game species in our study area.

Our estimate of animal biomass (10,190 kg/year) extracted in our study area suggests that wildlife remains an important component for the subsistence of rural people in the Yucatan Peninsula. About three quarters (7,998 kg) of that biomass came from just seven mammals (six ungulate species and the paca). This proportion is consistent with data found in previous studies across southeast Mexico [23,28]. Interestingly, the high numbers of turkeys and curassows hunted during this study contributed to a surprising 15.2% (1,552 kg) of total biomass taken by hunters. This figure contrasts with the modest proportion of bird biomass extracted (0.2-5.4% of total) during previous research done in southeast Mexico [7,8,24,28,58,61].

Seasonality seemed to be relevant for certain hunting practices in the Yucatan Peninsula. During the dry season, forest tracts constituted important hunting areas probably because of the presence of water bodies and abundant ripe fruits within them, as well as better access to remote hunting sites. In the rainy season, hunting activities substantially increased in agricultural areas, especially when corn and other crops were about to be harvested. We observed examples of seasonal hunting on pacas, which are easier to detect when they walk noisily on the forest litter during the dry season. That season is better to hunt singing males of ocellated turkeys and curassows as well. On the other side, the rainy season may be more appropriate to search for collared peccaries and white-tailed deer browsing on a variety of plants found in the *milpas*.

Jorgenson [7] did a pioneer study on wildlife biomass extracted in HXZ alone, recognizing himself biases in his estimates due to payments stimulating killings of abundant small species such as opossums and armadillos, which are considerably easier to hunt than large game. Our assessment of biomass extracted in HXZ + UHM (3,617.5 kg/year; N = 291) suggests that hunting remains an important activity in this community two decades after Jorgenson’s study. Methodological differences and factors such as the effects of hurricane *Dean* in 2007 make it difficult to contrast Jorgenson’s results with ours in terms of differences in animals and biomass harvested after two decades. However, we found a consensus of residents interviewed in XHZ + UHM and CHV in relation to a generalized decrease in game abundance on their lands within the last 20 years.

In Maya and Mestizo communities of the Lacandon Forest, Naranjo et al. [28] estimated a biomass of 8,160 kg/year extracted by hunters. Pacas, red brocket deer, and white-tailed deer were the most important prey species contributing 53% of total volume [28]. In our study, white-tailed deer, collared peccaries, and pacas contributed 65% of total biomass taken. Thus, the most important mammals in terms of biomass for hunters of the Lacandon Forest and the Yucatan Peninsula are the paca and the white-tailed deer, respectively [23,28].

### Final considerations

This study shows that subsistence hunting remains a frequent practice in rural communities of the Yucatan Peninsula, southeast Mexico. Unlike other regions of Mexico, Central and South America, where hunting has contributed to local extinctions of wildlife in densely populated areas [13,35,80], low human densities on extensive territories in our study area facilitate the conservation of large tracts of tropical forest in relatively good condition. These factors coupled with an increasing choice of productive alternatives and economic incentives (e.g., increasing tourism in the area, subsidies for rainforest and wildlife conservation, and creation of community reserves), have favored lowering hunting pressure on wildlife populations. However, if human population, economic development and land fragmentation keep growing fast in the Yucatan Peninsula, valuable game species will suffer overexploitation and habitat loss [23,36,49,59], which in turn might affect the subsistence of hunters and their families. For this reason, we are certain that this study contributes to better understand how subsistence hunting practices are changing at regional scale.

The proximity of our communities of study (wildlife sinks) to large protected areas (wildlife sources) probably attenuates negative effects on populations in spite of continuous hunting pressure. Nonetheless, potential wildlife source-sink systems may lead to assume local sustainable hunting when there is not [64,81]. Consequently, large-scale studies on hunting sustainability will be necessary to take proper decisions to conserve prey populations of the Yucatan Peninsula in the long-term [40,82].

Here we present a synthesis of the most relevant information about hunting practices gathered in the four communities visited during the study. Many variables that have not been analyzed remain to be discussed in further manuscripts that may provide light on complex environmental, cultural, and economic aspects of hunting in the tropical forest of the Yucatan Peninsula. In addition, reliable information on densities of game species will be needed for quantitative assessments on hunting sustainability in this region. Such assessments will be important for evaluating present and future risks on game species, particularly those that are already threatened or endangered (e.g., Ocellated Turkey, Great Curassow, white-lipped peccary –*Tayassu pecari–*, and Baird’s tapir –*Tapirus bairdii*).

Subsistence hunting needs to be better understood, revalued and regulated for the mutual benefit of prey populations and their users. Official incentives are necessary to encourage wildlife users to design, implement, and enforce their own hunting rules in their territories (e.g., spatial, temporal, numeric, and species-specific restrictions). Furthermore, it is clear that current laws regulating hunting in Mexico have gaps, especially regarding subsistence practices in poor communities. In our opinion, the federal government’s Wildlife General Law (*Ley General de Vida Silvestre*) [41] should be revised to clearly state what subsistence hunters can and cannot do, and how they may contribute to the sustainable use and conservation of a wide variety of game species in their own territories. In this sense, wildlife co-management strategies in communities of the Yucatan Peninsula may be a viable alternative that should be encouraged by environmental authorities [83,84]. Those strategies will be much benefited from quantitative and qualitative information on local wildlife uses, harvest rates, and traditional knowledge of indigenous and mestizo peoples.

## Consent

Written informed consent was obtained from the authorities (*comisarios ejidales*) of each community for conducting this research project and publishing this report and accompanying images.

## Competing interests

The authors declare that they have no competing interests.

## Authors' contributions

DSF wrote early drafts of the proposal and the manuscript, and did fieldwork in the study area between March 2010 and March 2011. EJN reviewed and improved the proposal and the manuscript, collaborated in the data analyses, and provided financial and logistical support for the study. JLR reviewed the manuscript and performed the statistical analyses. All authors read and approved the final manuscript.

## Supplementary Material

Additional file 1 **Appendix 1. **Hunting record form used in the communities visited during the study. Click here for file
