# Supplementary figures and images for: Wildlife uses and hunting patterns in rural communities of the Yucatan Peninsula, Mexico
Source: J Ethnobiol Ethnomed. 2012 Oct 2;8:38. doi: 10.1186/1746-4269-8-38 (PMC3508864; doi:10.1186/1746-4269-8-38)

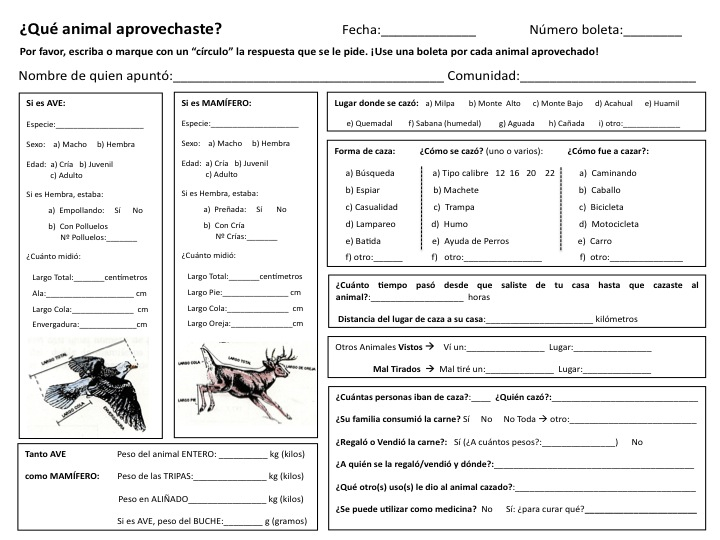

Supplement: Additional file 1 — Appendix 1. Hunting record form used in the communities visited during the study. [file 1746-4269-8-38-S1.jpeg]
